# Hydrophobic Silica Aerogel Thermal Protection for a Low-Power, Helium-3-Free Cosmic-Ray Neutron Soil Moisture Sensor in Hyper-Arid Rangeland

**DOI:** 10.3390/s26196266

**Published:** 2026-10-02

**Authors:** Nujud Badawi, Abdel Nasser Tawfik, Abousrie A. Farag, Azzah Aziz AlShehri

**Affiliations:** 1 Department of Physics, College of Science, University of Hafr Al Batin (UHB), Al Jamiah, Hafr Al Batin 39524, Saudi Arabia; nujuds@uhb.edu.sa; 2 Department of Physics, Islamic University of Madinah, Madinah 42351, Saudi Arabia; atawfik@acu.edu.eg; 3 Department of Agricultural Systems Engineering, College of Agricultural and Food Sciences, King Faisal University, Al-Ahsa 31982, Saudi Arabia; afaraj@kfu.edu.sa; 4 Department of Science and Technology, University College at Nairiyah, University of Hafr Al Batin (UHB), Nairiyah 31981, Saudi Arabia

**Keywords:** soil moisture, cosmic-ray neutron sensing, sensor design, thermal management, boron-10 lined detectors, hyper-arid monitoring

## Abstract

Soil moisture sensors in hyper-arid rangeland face temperatures beyond the range over which their electronics are specified, yet insulation placed around a neutron detector is a scatterer and risks being read as a change in soil water. We evaluate this trade-off computationally for a helium-3-free, boron-10-lined cosmic-ray neutron sensor fitted with an external hydrophobic silica aerogel sleeve. A voxel model of the instrument in a domain of its own size gives response functions, normalized to a maximum of 1.0 and applied to the spectrum of a 1000 m field simulation with no detector. A 20 mm sleeve raises the weighted epithermal count by 3.0%, constant to 0.0007 across a factor of six in soil water. The wet-to-dry scaling factor N(0.05)/N(0.30) is 2.1533±0.0161 bare and 2.1530±0.0160 sleeved, a ratio of 0.9999. The sleeve changes the count rate and not the moisture response, and the offset is absorbed into the calibration constant when calibration follows assembly. A diurnal thermal calculation gives an interior peak of 59.9 °C bare against a specified limit of 45 °C, and 40.7 °C sleeved. Every result is from simulation; field deployment remains future work.

## 1. Introduction

Soil moisture at the field scale is difficult to measure well. Point probes sample a few liters; satellite retrievals average over kilometers and penetrate only the top few centimeters. Cosmic-ray neutron sensing occupies the gap between them, integrating over several hectares and the upper few decimeters of the profile without contact with the soil [1,2,3]. Its use for irrigation management specifically is now supported by field trials across a range of settings, from open fields to small orchards [4,5,6,7], and by work relating the measurement to crop water-use efficiency [8]. Related developments extend the same signal to biomass quantification [9], to spatial scaling across networks [10,11], to multi-purpose observatory use [12], and to low-power telemetry architectures suited to remote sites [13]. The method exploits the inverse relationship between the near-surface epithermal neutron flux and the hydrogen content of the environment, of which soil water is normally the dominant part.

The case is strongest where water is scarce and irrigated areas are large. Forage production in the Arabian Peninsula draws heavily on groundwater, with alfalfa (*Medicago sativa* L.) commonly irrigated at 10,000–$20,000\;m^{3}\;{\mathrm{ha}}^{-1}\;{\mathrm{year}}^{-1}$ [14,15], and where soil moisture is strongly heterogeneous in space and time [16], favoring an integrating measurement over point sampling.

What makes unattended deployment feasible at all is the power budget. Boron-10-lined counters operate at far lower bias than helium-3 or BF_3_ tubes, and complete instruments of this class draw less than 0.5 W, with recent variants approaching 50 mW [17]. A load of that size is met by a small photovoltaic panel and buffer battery, which is how such systems are supplied commercially. The instrument therefore needs no mains connection, no fuel and no scheduled servicing, which is the precondition for monitoring rangeland where none of these exist. The same solar exposure that supplies that power also heats the instrument.

Two obstacles stand between the method and routine desert deployment. The first is supply: most installed CRNS instruments use helium-3, whose availability is constrained and whose cost has risen accordingly, so the field has moved toward helium-3-free alternatives [17,18,19,20]. The second is environmental. Summer air temperatures in the target rangeland reach 45 °C and surface temperatures exceed 50 °C, at or beyond the range over which detector electronics are specified. Gas-filled counters are not thermally indifferent: the gain of a proportional counter depends on gas density, and measurable degradation of counting performance with temperature has been documented for BF_3_ tubes [21], while detector development for high-temperature and irradiation-heavy environments remains an active problem [22]. Passive thermal protection is the obvious remedy. The difficulty is that any material placed around a neutron detector is itself a scatterer. An insulating sleeve that measurably moderates the incident spectrum would be read by the instrument as a change in soil hydrogen, that is, as a false moisture signal. The insulation must therefore be shown to be radiometrically transparent before it can be adopted, and that is a simulation question rather than a thermal one.

The question is addressed here with the Monte Carlo transport code URANOS [23,24], applied to a StyX S2 boron-10-lined proportional counter fitted with an external aerogel sleeve. The work makes three contributions. First, it quantifies the radiometric effect of the sleeve from a voxel model of the instrument and a response function computed from it. Second, it measures the effect on the moisture response itself rather than on the count rate alone, which is the quantity that determines whether the instrument remains usable. Third, it establishes that the resulting offset is constant across the moisture range and is therefore absorbed into the instrument’s calibration constant. All results are obtained by simulation. Instrument dimensions, material densities, thermal conductivities, detector efficiencies and site climate normals are taken from the manufacturer’s documentation and from the published literature and are identified as such where they are used; no measurement was performed for this work. Field deployment and calibration are outside the present scope.

## 2. Background and Detector Selection

Fast neutrons produced in atmospheric cascades lose energy by repeated elastic and inelastic scattering until, near the ground, a substantial fraction lies in the epithermal band between roughly 0.5 eV and 100 keV [25,26]. Hydrogen is by far the most efficient moderator per unit mass, because a neutron can lose its entire energy in a single collision with a proton. The epithermal flux above a soil surface is therefore governed principally by the hydrogen inventory beneath it, and the measured count rate falls as soil water rises [1].

Converting a count rate to water content requires the raw counts to be corrected for the atmospheric and heliospheric variability that also modulates the flux, and then inverted through a calibrated relation. Both steps are established and are summarized in Section 4. What is site-specific is the spatial weighting: the measurement is not uniform over its footprint but declines with radial distance, and the weighting function itself contracts as the soil becomes wetter [1,27]. Under dry conditions, the 86% footprint radius reaches 200 m or more, which is why the simulation domain has to be large.

Three detector families are practical for CRNS. Table 1 summarizes the comparison that led to our choice. Helium-3 counters remain the reference for intrinsic efficiency but are constrained by isotope supply. Boron trifluoride counters are efficient and inexpensive, but BF_3_ is acutely toxic, with a ceiling exposure limit of 1 ppm and an IDLH of 25 ppm, and it hydrolyses in moist air to boric acid, hydrogen fluoride and fluoboric acid [28,29], which is disqualifying for an instrument sited among livestock in open rangeland: a pressure-vessel failure would release a toxic gas into a grazing area with no supervision. Boron-10-lined counters convert neutrons in a thin solid ^10^B_4_C film deposited on the tube wall and are filled with an inert Ar:CO_2_ mixture. Their per-tube efficiency is lower, which is compensated in practice by multiplying the coated area, and they carry neither the supply constraint nor the toxicity hazard [17,20]. Comparative measurements against helium-3 counters bear this trade-off out [30], and the architecture is mature enough to be available commercially [31,32]. Broader reviews of gas-based neutron detector development [33] and of helium-3 alternatives [34] reach similar conclusions. Two long-term considerations favor it further: helium accumulation and lattice damage in the converter film are slow enough to be immaterial over an instrument lifetime [35], and space-charge effects in proportional counters are well characterized [36]. We adopt the StyX S2, a three-tube boron-10-lined instrument designed for autonomous field operation. Boron-10-lined counters trade some intrinsic efficiency for a solid, non-toxic converter, and have been developed specifically as drop-in replacements for helium-3 tubes [37].

## 3. Instrument and Simulation Setup

The detector response and the field neutron transport are computed in two separate stages. The first builds a voxel model of the StyX S2 in a domain the size of the instrument and returns its energy-dependent detection efficiency. The second simulates the neutron field over a 1000 m domain containing no detector. The response functions from the first stage weight the spectrum recorded in the second. All transport is performed with URANOS v1.35 [23,24].

### 3.1. Detector Model

The base unit is a high-density polyethylene cylinder 146 cm long and 28 cm in diameter with a wall thickness of 2.4 cm, containing up to three boron-lined counter tubes of 125 cm length in an Ar:CO_2_ fill [38]. The instrument carries a gadolinium-based thermal neutron shield as an external mantle [17].

The model is built as a stack of horizontal layer maps at 0.5 mm pixel pitch in a 400×400 mm domain. The cylinder axis lies in the horizontal plane, so each map is a set of strips spanning the domain and the circular cross-section is assembled from the chords of successive layers. This is the construction used in the URANOS reference detector examples. Laying the instrument down makes illumination from above equivalent to a transverse crossing of the instrument, which is the geometry a vertical detector presents to the majority of an approximately isotropic field. The response so obtained is that of a transverse crossing. It does not represent axial incidence, in which a neutron entering the end of an upright instrument traverses 1.46 m of moderator, and it resolves no angular dependence.

Thirteen slices cover the 281 mm stack. The central slice is 50 mm thick and contains the whole counter tube, since URANOS admits a single detector layer. The twelve flanking slices are 13.42 mm each. A 5 mm cap layer is inserted above and below every slice; in each cap the gadolinium occupies the annulus between the chords of the two adjacent slices, which is the horizontal ledge of the stepped surface at that height. Without these caps, a neutron falling from above travels parallel to the shield and does not cross it. With them, the vertical path through the mantle is 100μm of Gd_2_O_3_ equivalent at every lateral offset inside the casing, 50μm on entry and 50μm on exit.

The mantle is drawn one pixel thick at 10% density in the slices and 5 mm thick at 1% density in the caps. Both give the areal density of the physical shield, near 50μm of Gd_2_O_3_. Absorption follows exp(−nσt) and depends on the product of number density and thickness alone, so it is reproduced exactly whatever the optical depth; for the areal density used here that depth is 6.0 at 25 meV and falls below 0.1 in the epithermal range, gadolinium being strongly non-1/v above its resonance. Scattering needs the further condition that the layer be thin, since only then is the first-collision probability likewise a function of nt. The scattering optical depth is 0.021 and the multiple-scattering correction inside the layer is of order $\tau^{2}/2$.

Following the procedure suggested in the review, the boron converter film is replaced by a volumetric helium-3 absorber. Detection is then proportional to the path length of a neutron in the gas and carries no dependence on how a micrometer-scale coating is discretized on a 0.5 mm grid. A single 50 mm tube is placed on the axis, with a 1 mm HDPE wall.

**Figure 1:**
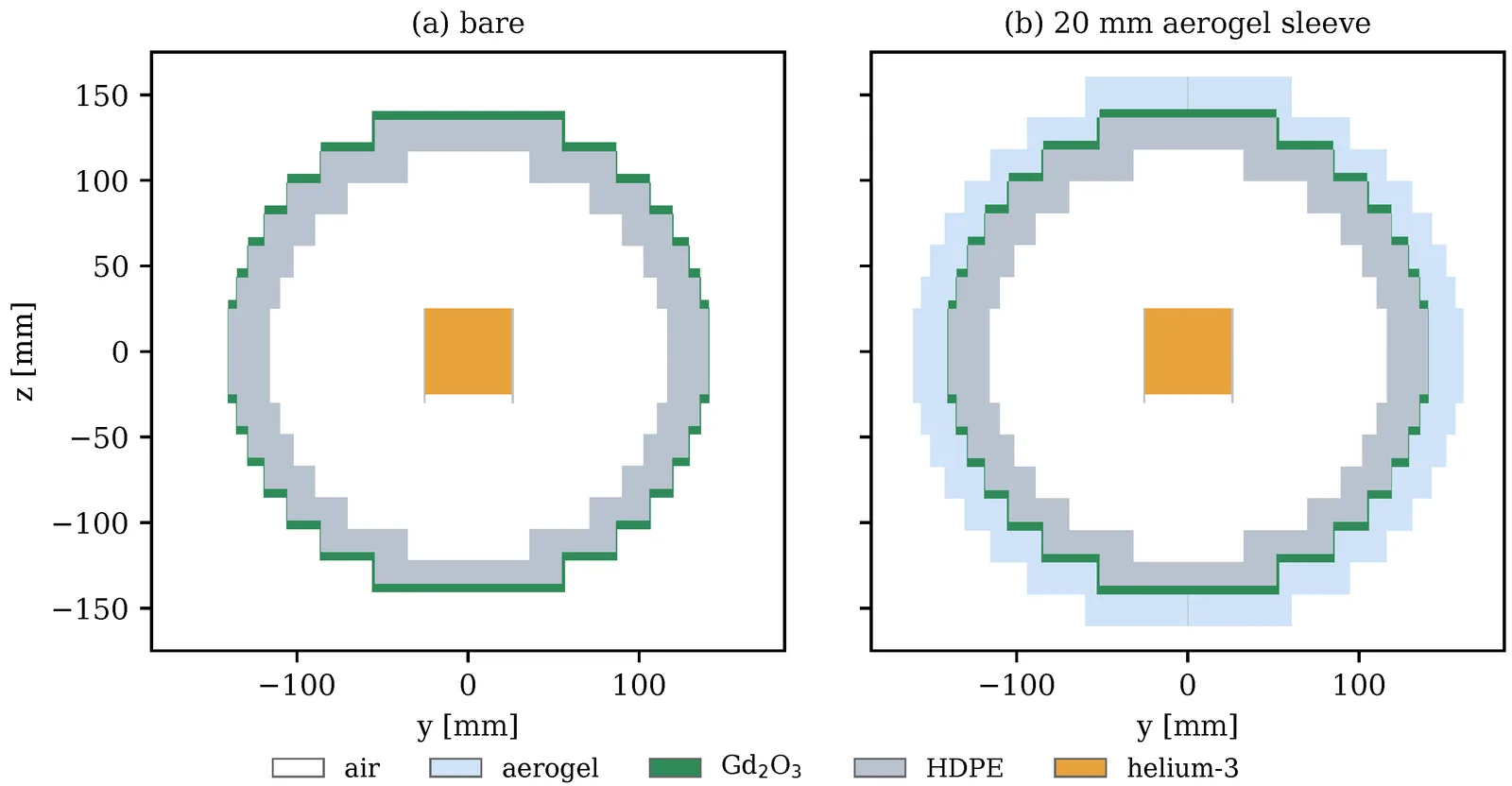
Cross-section of the modeled instrument in the plane perpendicular to the tube axis, rendered directly from the layer maps used in the simulation. The gadolinium mantle lies outside the HDPE wall. The stepped outline is the discretization of the circular cross-section into thirteen horizontal slices; the thin horizontal segments of mantle are the cap layers that shield the ledges of that outline. The aerogel sleeve of panel (**b**) is the only difference between the two configurations.

Figure 1 shows the cross-section of both configurations as modeled. The sleeved configuration adds a 20 mm annulus of silica aerogel outside the mantle, represented as quartz at 8% density, 0.20 g cm^−3^. It extends the stack to 321 mm and adds one slice and one cap at each end. The thirteen slices covering the casing are pixel-identical between the two configurations, and the casing center sits at the same height in both.

### 3.2. Aerogel Sleeve

The sleeve represents a commercial flexible silica aerogel blanket (Pyrogel XTE, Aspen Aerogels, Northborough, MA, USA), chosen over monolithic aerogel for mechanical robustness under handling and thermal cycling [39,40]. The manufacturer specifies a bulk density of $0.20\;g\;{\mathrm{cm}}^{-3}$ and a thermal conductivity of $20\;\mathrm{mW}\;m^{-1}K^{-1}$. An intra-laboratory study characterized twelve insulating materials by three independent techniques and reported 13–$20\;\mathrm{mW}\;m^{-1}K^{-1}$ for commercial aerogel insulation, placing the declared value at the least favorable end of that interval [41]. That study could not characterize thin aerogel blankets by the transient method at all, the specimens being too thin for the heat front to remain within them, so the thermal characterization identified as future work should be planned around steady-state measurement [42].

The blanket is a composite: 30–40% amorphous silica by mass, 40–50% glass-fiber reinforcement and 1–5% Al(OH)_3_ as opacifier. It is not hydrogen-free. Taking the upper bound of the Al(OH)_3_ fraction and its stoichiometric hydrogen content gives about 0.19% hydrogen by mass; with the methyl groups of the hydrophobic surface treatment [43] we adopt 0.2% as a bound. HDPE is 14.3% hydrogen by mass. The simulation represents the sleeve as quartz scaled to the blanket density, which reproduces its silica scattering but carries no hydrogen.

Aerogel blanket is not used bare outdoors. Ultraviolet exposure, rain and freezing degrade the fiber facing and the hydrophobic surface treatment, and the material is supplied for service under a jacket for that reason: the manufacturer’s installation guidance treats metal jacketing as part of the insulation system and states that protection begins once the installed blanket has been jacketed [44]. The sleeve is therefore deployed inside a weather jacket, which also fixes the solar absorptivity of the outer surface and so enters the thermal calculation of Section 6. The rate at which the hydrophobic treatment deteriorates under combined thermal and solar loading has been measured [45], and accelerated aging of the jacketed assembly is listed among the work required before field application.

### 3.3. Computation of the Response Functions

Response functions are computed with the URANOS detectorBatchRun2 option, which launches a fixed number of monoenergetic neutrons at each of 40 energies spaced logarithmically from $1.02\times 10^{-8}$ to 57.5 MeV. Each energy uses $2\times 10^{5}$ neutrons. The source is a surface of the same lateral extent as the domain, placed 0.5 m above the instrument. A volume source generates neutrons inside the instrument itself, which bypass the shield and the moderator; with a volume source, the thermal fraction of the response is 16.7% and its maximum falls at the lowest energy sampled.

Thermal neutron transport is enabled. Without it, URANOS removes neutrons on reaching the thermal range and the three lowest energies return no counts.

Each response function is normalized so that its largest efficiency equals 1.0. The normalized functions are given in the Supplementary Material.

### 3.4. Field Simulation Setup

The field stage uses a 1000 m domain with a 499 m volume source, a 3 m soil layer and no detector geometry. Counts are scored in a vertical cylinder of 150 m radius over the interval $10^{-9}$ to 19 MeV. Each run launches $2\times 10^{6}$ neutrons. Soil solidity is 0.50, air humidity 2.333 g m^−3^, atmospheric depth 1013 g cm^−2^ and cutoff rigidity 14.5 GV. A volume source is used here, unlike in the detector stage: the field domain contains no detector geometry, so no neutron is generated inside an instrument and none bypasses a shield or a moderator. Three volumetric water contents are simulated: 0.05, 0.15 and 0.30. Thermal transport is enabled here as well, so the recorded spectrum extends to $2\times 10^{-9}$ MeV rather than terminating near $5\times 10^{-8}$ MeV.

The incident spectrum is sampled from the analytical parameterization of the atmospheric cascade by Sato and Niita [46], as distributed with URANOS, and is scaled by the atmospheric depth and cutoff rigidity above. The conversion from incident flux to the flux returning from the ground is not parameterized: each neutron is transported through air and soil and its history followed. The cutoff rigidity of 14.5 GV is the value reported for the cosmic-ray station at Riyadh, the nearest reference site to the target rangeland, against 14.8 GV at Jeddah [47]; it is substantially above the 5 GV used as a standard value in mid-latitude CRNS simulations [48] and admits only more energetic primaries, which hardens the incident spectrum.

The scoring cylinder is a numerical volume and not a physical component. Because the soil is laterally homogeneous, enlarging its radius averages over many identical co-located instruments and reduces counting-statistics uncertainty without altering the physics sampled, which is the established convention in URANOS-based CRNS work [48].

### 3.5. Weighting and the Wet-to-Dry Scaling Factor

The spectrum recorded in each field run is weighted bin by bin by the normalized response function, interpolated linearly in logE. This yields, for each soil water content and each configuration, the count a detector with that response would register in that field. The quantity compared between configurations is the wet-to-dry scaling factor $N\left(\theta_{1}\right)/N\left(\theta_{2}\right)$ computed within each configuration, not the ratio of one configuration to the other at fixed θ.

Both response functions are applied to the same three field spectra. The sampling fluctuations of those spectra are therefore common to the two configurations and cancel in the ratio of their scaling factors, which is quoted with the individual quotients in Section 5.

### 3.6. Model Assumptions

Table 2 lists the simplifications in the detector model and their effect on the reported quantities.

## 4. Correction Framework

Raw epithermal counts are converted to a corrected rate through the standard multiplicative chain,

(1) $$N_{\mathrm{final}}=N_{\mathrm{raw}}\cdot f_{p}\cdot f_{h}\cdot f_{c},$$

where $f_{p}$ corrects for atmospheric pressure, $f_{h}$ for atmospheric humidity, and $f_{c}$ for incoming cosmic-ray intensity [49,50]. The corrected rate is then inverted to soil water content through a calibration relation whose constant, $N_{0}$, is determined once for a given instrument at a given site. This constant absorbs any multiplicative offset introduced by hardware fitted to the instrument.

## 5. Results

### 5.1. Response Functions

**Figure 2:**
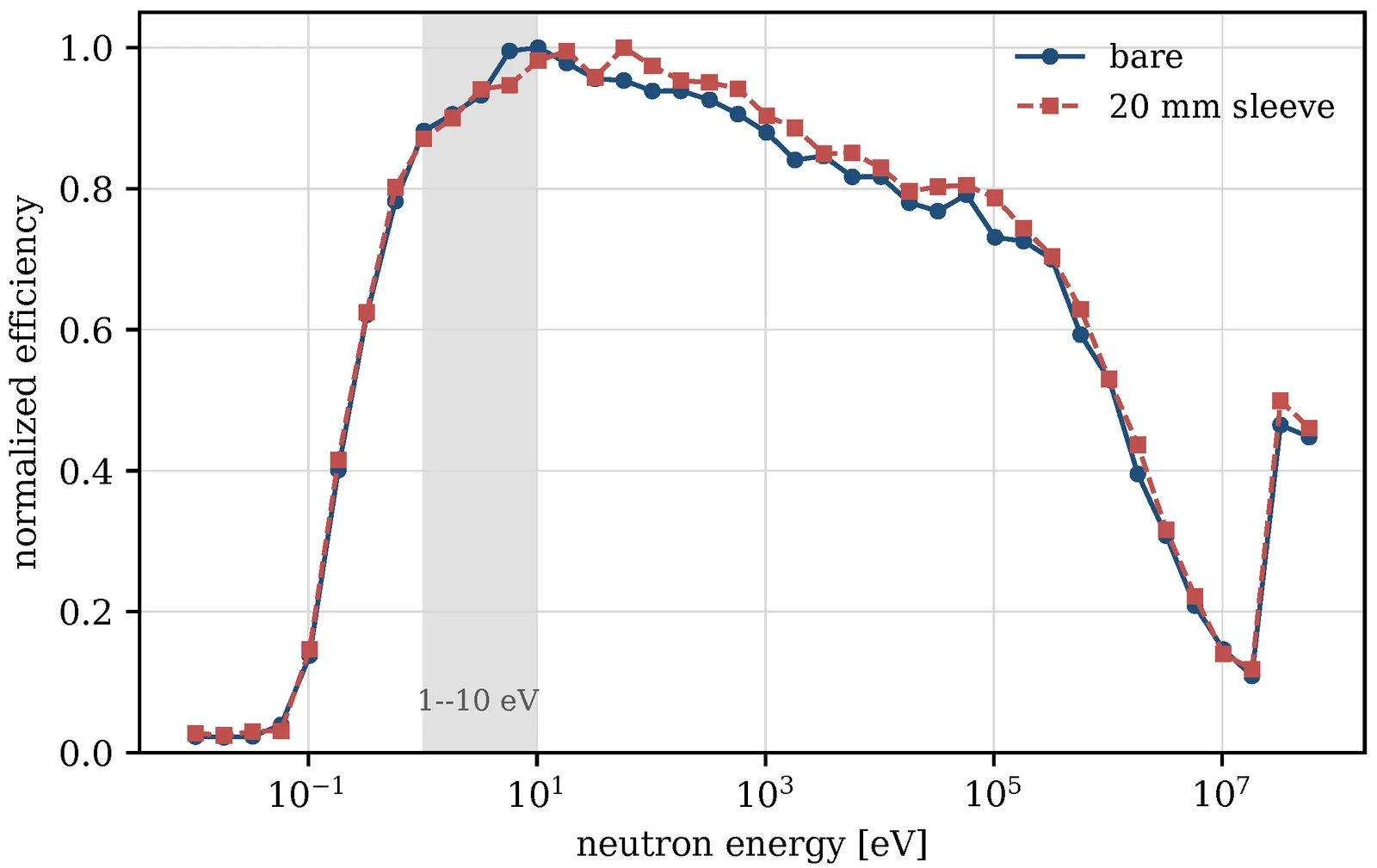
Response functions of the two configurations, normalized so that the largest efficiency equals 1.0. The shaded band marks the 1–10 eV interval in which detectors of this class have their maximum [51]. The rise above 10 MeV comes from the high-energy cascade model. It contributes 0.8% of the weighted field count, so it does not affect the comparison.

Table 3 and Figure 2 summarize the two response functions. Both rise from the thermal range, reach a maximum in the epithermal decade and fall towards 1 MeV. The maximum of the bare function lies at 10.2 eV, within the 1–10 eV interval reported for detectors of this class [51]. The maximum of the sleeved function lies at 57.5 eV. In each function the largest point exceeds its immediate neighbors by one to two standard deviations, so on a grid of 40 energies the position of the maximum is not determined to better than a decade.

The peak efficiency of 2.12% is a property of the model and of the source geometry, not of the instrument. It is the probability that a neutron launched from the source plane is captured in the helium-3 volume, and it is set by the size of that volume relative to the source area and by the substitution of a volumetric absorber for the boron film. It is not comparable with the 9–10% efficiency quoted for a physical counter at 25 meV [17], which is referred to thermal neutrons incident on the counter itself. Every quantity reported below is a ratio between configurations that share this geometry.

The thermal fraction of the bare model is 0.270%. For the physical shielded S2 the corresponding figure is 1–3%, against 15% for an unshielded instrument [17]. The modeled mantle therefore suppresses the thermal component in the right direction and somewhat more strongly than the physical one. The stepped construction gives every vertical path the same 100μm of Gd_2_O_3_ equivalent, whereas a cylindrical shell presents a path that lengthens towards grazing incidence.

Summed over all 40 energies, the sleeved efficiency is 1.9±0.4% lower than the bare one. After normalization to a peak of 1.0, the ordering reverses, and the weighted counts of Section 5.3 are higher for the sleeved configuration by 3.0%. The cause is the value of the maximum, not its position. Normalization divides each function by its own peak efficiency, 2.115% bare and 2.029% sleeved. The sleeved peak is lower by a factor of 1.042 while its summed efficiency is lower by a factor of 1.020, so dividing by the peak lifts the sleeved curve above the bare one by 2.2% over the range as a whole, and by 3.0% once weighted by a field spectrum concentrated in the epithermal decade. Both numbers are reported here because they answer different questions. The summed efficiency describes how many neutrons the modeled detector registers; the normalized function is what the two-stage procedure requires and is what enters the weighting.

### 5.2. Field Simulation

**Figure 3:**
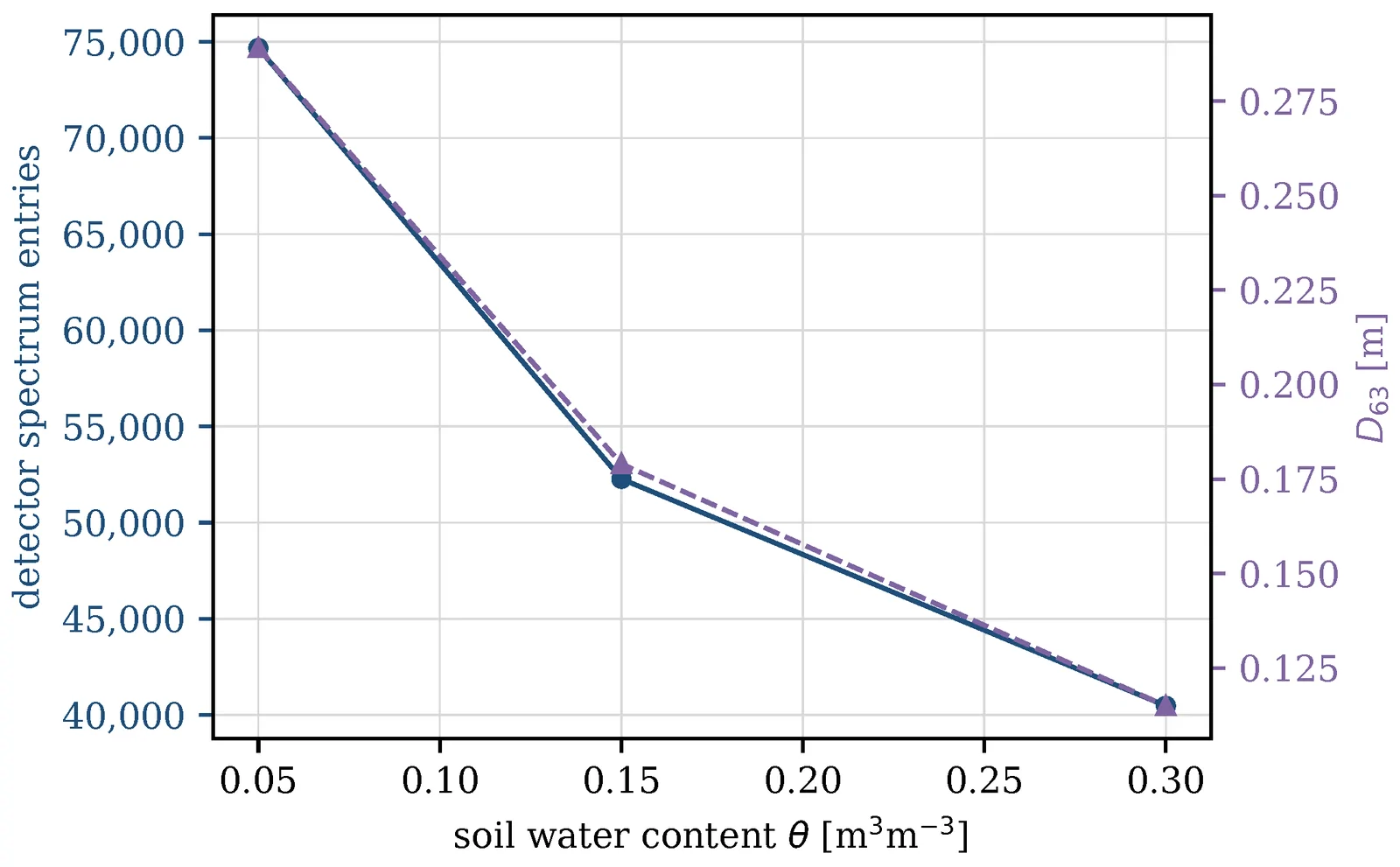
Field simulation with no detector present. Left axis, entries in the recorded detector spectrum against soil water content. Right axis, the effective sensing depth $D_{63}$, which contracts by a factor of 2.5 across the range as added hydrogen shortens the neutron mean free path.

Table 4 and Figure 3 give the field runs. The recorded spectrum extends from $2\times 10^{-9}$ to $7\times 10^{3}$ MeV. Counts fall by a factor of 1.85 between θ=0.05 and θ=0.30, and the fall is non-linear: the decrease over the first $0.10\;m^{3}m^{-3}$ exceeds that over the following 0.15.

The sensing depth contracts from 0.289 to 0.115 m across the moisture range, as added hydrogen shortens the neutron mean free path.

The horizontal footprint is not reported. Counts are scored throughout a cylinder of 150 m radius, so the distance between the point at which a neutron last left the soil and the point at which it was scored carries the extent of that cylinder as well as the transport distance, and the percentiles obtained from it do not vary with soil water content. A footprint determined this way would not be comparable with the 147–273 m of Brogi and coworkers [4], which refers to an instrument of finite size, and it would contradict the contraction with wetness established for this measurement [1,27]. Determining it here would require a point detector and is outside the scope of this comparison.

### 5.3. Weighted Comparison and the Wet-to-Dry Scaling Factor

**Figure 4:**
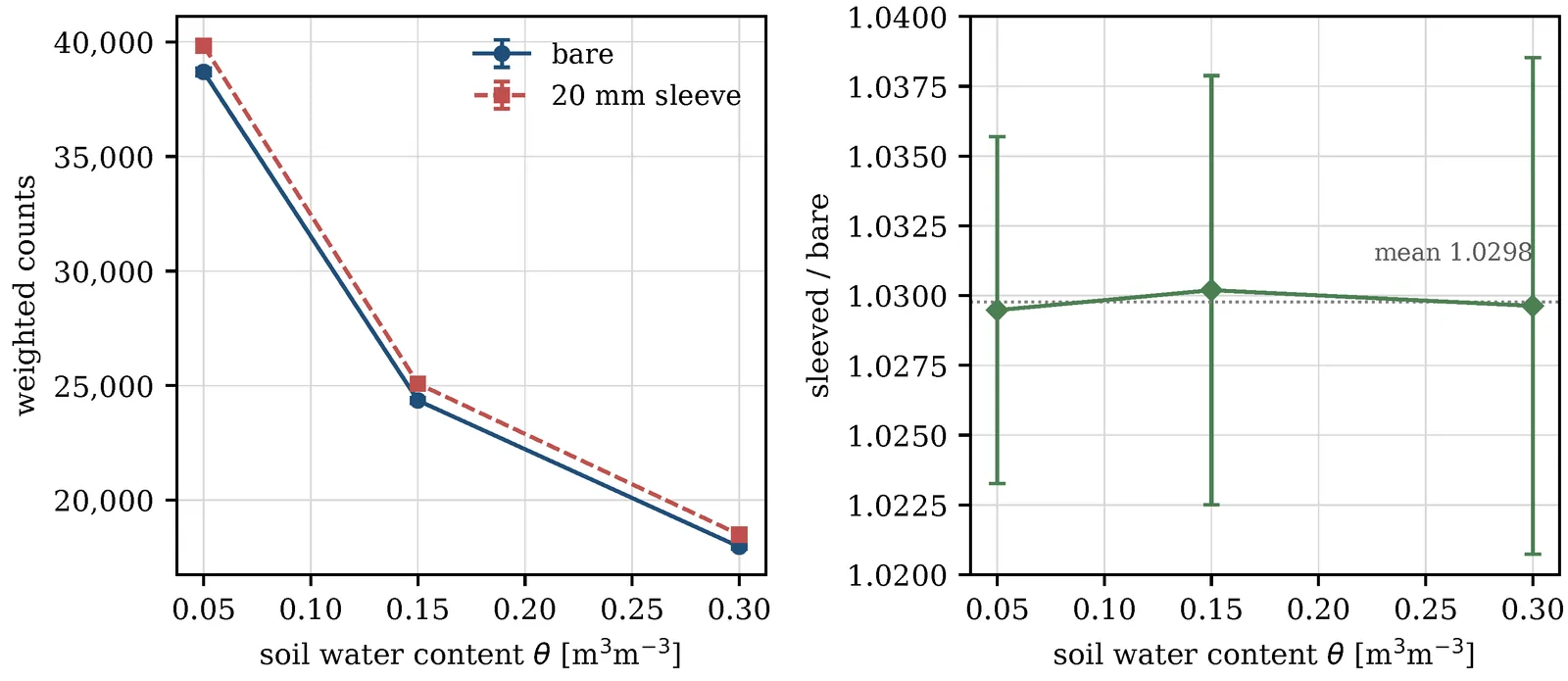
(**Left**) field spectra weighted by the two normalized response functions. (**Right**) the ratio between them at each soil water content, with the mean shown as a dotted line. The ratio varies by 0.0007 across a factor of six in soil water.

Weighting each field spectrum by each normalized response function gives the counts in Table 5 and Figure 4. The sleeve raises the count by 2.95, 3.02 and 2.96% at the three moisture levels, a spread of 0.0007 across a factor of six in soil water.

The quantity that determines whether a modified instrument remains usable is the wet-to-dry scaling factor computed within each configuration (Table 6).

The uncertainties quoted on the individual scaling factors are those a single measurement would carry. The ratio between the two configurations is more precisely determined than they are, because both response functions are applied to the same three field spectra: the sampling fluctuations of those spectra are common to the two configurations and cancel in the ratio. The ratio of scaling factors is 0.99986 for N(0.05)/N(0.30) and 0.99931 for N(0.05)/N(0.15).

A multiplicative offset independent of soil water is absorbed into the calibration constant $N_{0}$ of Equation (1). An instrument calibrated with its sleeve fitted returns the same soil water content as one calibrated without it, and a sleeve retrofitted to a calibrated instrument shifts the reported count rate by 3% until $N_{0}$ is redetermined.

## 6. Thermal Performance

The sleeve is fitted for a thermal purpose that URANOS does not model. This section gives a one-dimensional calculation of that purpose with stated inputs. It is a prediction, not a measurement.

The base unit is a sealed passive enclosure. The amplifier, the logger, the battery and the charge controller are housed separately and connected by cable [38], so the cylinder contains no internal heat source. For such a body, a steady-state calculation is uninformative: insulation resists inward and outward flow equally, and at steady state the interior approaches the sol-air temperature whatever the thermal resistance. What insulation changes is the amplitude of the diurnal cycle. The casing is treated as a single lumped capacitance *C* behind a total resistance *R*, driven by a sinusoidal sol-air temperature of mean $T_{m}$ and amplitude *A*, giving an interior peak of

(2) $$T_{\max}=T_{m}+\frac{A}{\sqrt{1+{\left(\omega \tau \right)}^{2}}},\;\tau =RC,$$

with ω=2π/24 h^−1^.

The HDPE casing has a mass of 26.8 kg and a heat capacity of 50.9 kJ K^−1^; with the tubes and hardware C=55 kJ K^−1^. Site inputs are a peak global horizontal irradiance of 1100 W m^−2^, a peak summer air temperature of 45 °C and a mean August minimum of 27.3 °C. The solar elevation at the summer solstice is 85° at this latitude, and with a desert albedo of 0.35 the area-averaged incident flux at noon is 320 W m^−2^. The combined external film coefficient is 16.6 W m^−2^K^−1^, of which 10 is convective and 6.6 radiative. Thermal conductivities are 0.45 for HDPE and 0.020 W m^−1^K^−1^ for the aerogel blanket.

The two configurations do not present the same surface to the sun and the sky, and the solar load follows the surface rather than the insulation behind it. The bare instrument presents its black polyethylene casing, for which we take a solar absorptivity of 0.93 and a long-wave emissivity of 0.90. The sleeved instrument presents the jacket that an aerogel blanket requires for outdoor service, and that jacket is a specified component rather than an assumed property: we take a light or aluminized facing of absorptivity 0.40 at the same emissivity. Absorptivity enters the sol-air temperature directly, so over part of the range this distinction matters more than the insulation, and it is treated as a design variable in the sensitivity below.

Table 7 gives the resistance, time constant, damping factor and interior peak for each sleeve thickness.

Without a sleeve, the calculated interior peak is 59.9 °C, fifteen degrees above the specified upper limit. A 20 mm sleeve with a light jacket brings it to 40.7 °C. Two effects combine: the jacket cuts the absorbed solar flux, and the insulation damps the diurnal amplitude by a factor of 3.3 and shifts the interior maximum by about five hours. Neither reduces the daily mean.

The result is sensitive to the surface and to the wind, and the bounds are these. Raising the jacket absorptivity from 0.40 to 0.60 moves the sleeved peak from 40.7 to 42.7 °C, and to 44.7 °C at 0.80; lowering it to 0.25 gives 39.3 °C. In still air, $h_{\mathrm{conv}}=5$ W m^−2^K^−1^, the bare peak rises to 66.8 °C and the sleeved peak to 42.3 °C. On a record day for the region, 48.9 °C maximum with a 32 °C night, the bare peak is 63.8 °C and the sleeved peak 44.9 °C. The sleeved configuration leaves the rated range only where a dark jacket, still air and a record day coincide, which gives 49.3 °C.

The claim here is not that the instrument fails without a sleeve. The same authors report stable pulse-height and pulse-length discrimination for this electronics family over −10 to +55 °C, with drift coefficients of 0.046 and −0.02 units per degree, an order of magnitude below the width of the neutron discrimination region [17]. The sleeve keeps the instrument inside its rated envelope and reduces the amplitude of the daily thermal cycle it undergoes.

The prediction is testable at deployment without additional hardware. The logger writes internal and external temperature to envlog.txt and the amplifier temperature to hvlog.txt at the configured averaging interval [38].

## 7. Deployment Implications

A single sensor of this class integrates over roughly ten hectares [1], so one instrument reports the root-zone water content of a whole management unit. At under 0.5 W it runs from a small photovoltaic panel and buffer battery and requires neither mains supply nor scheduled servicing.

The sleeve raises the weighted count rate by 3.0% and leaves the wet-to-dry scaling factor unchanged to within 0.01%. Calibration performed on the assembled instrument absorbs the offset. Retrofitting a sleeve to a calibrated instrument without redetermining $N_{0}$ introduces a systematic error of 3% in count rate.

The thermal calculation of Section 6 selects the thickness. The radiometric result places no constraint on it: 20 mm and the bare configuration differ by 3% in count rate and by nothing in moisture response. On a typical summer day 10 mm would suffice, at 43.5 °C. It does not survive the adverse cases: 47.6 °C on a record day and 45.4 °C in still air, against 44.9 and 42.3 °C for 20 mm. Twenty millimeters is the thinnest sleeve that stays inside the rated range in both, and it is adopted on that basis.

Nothing in the argument depends on the soil being dry. The constancy of the offset was established across a factor of six in soil water, and any deployment facing a wide diurnal temperature range meets the same design problem.

### Limitations

The study is computational. No bench measurement was made, and the 3% offset is large enough for a bench test to be feasible. The detector model carries the simplifications of Table 2, of which the helium-3 absorber, the single 50 mm tube and the stepped cross-section affect absolute efficiency and are identical in the two configurations compared. The sleeve is simulated as quartz scaled to the blanket density, which reproduces its silica scattering but contains no hydrogen; the residual 0.2% hydrogen fraction of the physical blanket is not represented. Its areal hydrogen density, $8\times 10^{-4}$ g cm^−2^ over 20 mm, is a factor of 400 below the 0.33 g cm^−2^ of the HDPE wall immediately inboard of it. The weather jacket is not represented either: at 0.1 mm of aluminum it carries 0.027 g cm^−2^, eighty-four times less than the HDPE wall, contains no hydrogen, and scatters fewer than one neutron in a thousand. The thermal calculation is one-dimensional. Its two sensitive inputs are the convective coefficient, assumed at 10 W m^−2^K^−1^, and the solar absorptivity of each outer surface, 0.93 for black polyethylene and 0.40 for the jacket; both are bounded in Section 6.

The step from measurement to water saving remains open. Continuous root-zone moisture at field scale is an input to irrigation scheduling, and quantifying the volume of water a given strategy conserves requires paired field trials over at least one season.

## 8. Conclusions

A 20 mm hydrophobic silica aerogel sleeve raises the weighted epithermal count rate of a boron-10-lined cosmic-ray neutron sensor by 3.0%, constant to 0.0007 across soil water contents from 0.05 to $0.30\;m^{3}m^{-3}$. The wet-to-dry scaling factor N(0.05)/N(0.30) is 2.1533±0.0161 without the sleeve and 2.1530±0.0160 with it, a ratio of 0.9999. The sleeve changes the count rate and not the moisture response, and the offset is absorbed into the calibration constant when calibration follows assembly.

The result was obtained with a two-stage procedure: a voxel model of the instrument in a domain of its own size, response functions from detectorBatchRun2 normalized to a maximum of 1.0, and a field simulation in a 1000 m domain with no detector present, whose spectrum the normalized functions weight. An earlier version of this work computed the response functions with the instrument standing, which extruded the converter into a vertical column along the axis and returned the geometric shadow of that column rather than a detection probability. The measured plateau efficiency in that configuration, 0.206%, matched the projected area fraction of the column, 0.292%, and no shield or sleeve placed around the instrument altered it.

The thermal calculation gives an interior peak of 59.9 °C without a sleeve against a specified limit of 45 °C, and 40.7 °C with a 20 mm sleeve and a light jacket, by cutting the absorbed solar flux and damping the diurnal amplitude by a factor of 3.3. This is a calculation with stated assumptions and is testable from the logger’s own temperature record at deployment.

Work required before field application includes direct thermal characterization of the sleeve, which should be planned around steady-state rather than transient measurement and should include accelerated aging, since the hydrophobic surface treatment degrades under combined thermal and solar loading [45]; a bench measurement of the 3% offset; an explicit hydrogen-bearing material definition to replace the scaled quartz; and field deployment over at least one season. 

## Figures and Tables

**Table 1:** Comparison of the three practical CRNS detector technologies. The decisive criteria for an unattended rangeland deployment are supply security and the absence of a toxic inventory.

	^3^He	BF_3_	^10^B-Lined
Intrinsic efficiency	high	high	moderate
Isotope supply	constrained	available	available
Toxic inventory	none	severe	none
Operating voltage	moderate	high	low
Suitability here	limited	excluded	selected

**Table 2:** Simplifications in the detector model and their effect. Quantities reported in this work are ratios computed within and between configurations built from identical geometry apart from the aerogel.

Simplification	Reason	Effect
Helium-3 volumetric absorber in place of the ^10^B_4_C film	Removes the dependence of efficiency on how a 1.5μm coating is discretized on a 0.5 mm grid	Absolute efficiency is not that of the instrument; identical in both configurations
Instrument modeled lying down	Makes top-down illumination equivalent to a transverse crossing, the dominant case for a vertical detector in an approximately isotropic field	Response is that of a transverse crossing; axial incidence and the angular dependence are not resolved
Circular cross-section built from horizontal slices	Layer maps in URANOS are horizontal	Vertical path through the HDPE wall is short by 11% on average, from comparison of the stepped stack with the exact annulus at 0.5 mm intervals across the cross-section; identical in both configurations
One central tube of 50 mm diameter	The manufacturer’s manual gives tube length but neither diameter nor position; 50 mm is taken from the URANOS reference example	Absolute efficiency, and possibly a small shift in the energy of the maximum, since the moderator thickness in front of the gas differs; identical in both configurations
Tube wall as HDPE; copper converter foil not represented	Copper has no material code in URANOS	Absolute efficiency only
Mantle drawn thicker than 50μm at reduced density	50μm is below the pixel pitch	Areal density preserved; absorption is exact and the layer is thin enough for the equivalence to hold for scattering
400 mm section of a 1250 mm tube	Domain size	Modeled length identical in both configurations

**Table 3:** Response functions from detectorBatchRun2, $2\times 10^{5}$ neutrons at each of 40 energies. The thermal fraction is the sum of the three lowest energy points, 1.02 to $3.24\times 10^{-8}$ MeV. On a grid of 40 energies, the position of the maximum is not resolved beyond a decade.

	Bare	20 mm Sleeve
Summed counts	106,847	104,795
Peak efficiency	2.12%	2.03%
Energy of maximum	10.2 eV	57.5 eV
Thermal fraction	0.270%	0.319%

**Table 4:** Field simulation with no detector present, $2\times 10^{6}$ primary neutrons per run, scoring cylinder of 150 m radius. $D_{63}$ is the 63rd percentile of the maximum penetration depth of neutrons that entered the soil and subsequently re-crossed the surface.

θ [m^3^m^−3^]	Spectrum Entries	$D_{63}$ [m]
0.05	74,673	0.289
0.15	52,274	0.179
0.30	40,472	0.115

**Table 5:** Field spectra weighted by the normalized response functions. Uncertainties are propagated from the Poisson counts of the field spectra.

θ [m^3^m^−3^]	Bare	20 mm Sleeve	Sleeve/Bare
0.05	38,689±165	39,830±170	1.0295
0.15	24,352±129	25,088±132	1.0302
0.30	17,967±110	18,500±113	1.0296

**Table 6:** Wet-to-dry spectra weighted by the normalized response functions scaling factors within each configuration, and the ratio between them.

	Bare	20 mm Sleeve	Ratio
N(0.05)/N(0.30)	2.1533±0.0161	2.1530±0.0160	0.99986
N(0.05)/N(0.15)	1.5887±0.0108	1.5876±0.0108	0.99931

**Table 7:** Diurnal thermal model. The manufacturer specifies an operating range of −20 to +45 °C [38]. The winter minimum recorded for the region is −2.8 °C, so only the upper limit is in question. The bare row uses the absorptivity of black polyethylene, 0.93; the sleeved rows use the jacket value, 0.40.

Sleeve	*R* [m^2^K W^−1^]	τ [h]	Damping	Interior Peak [°C]
none	0.114	1.2	0.95	59.9
5 mm	0.364	4.0	0.69	46.3
10 mm	0.614	6.7	0.49	43.5
15 mm	0.864	9.4	0.38	41.8
20 mm	1.114	12.2	0.30	40.7

## Data Availability

The Supplementary Archive contains, for each of the two configurations, the complete set of material layer maps (2.png–28.png bare, 2.png–32.png sleeved), the corresponding density layer maps (2d.png–28d.png and 2d.png–32d.png) and the URANOS geometry file UranosGeometryConfig.dat. It also contains make_layer_maps.py, which generates every one of those files from the dimensions given in Section 3, and the two normalized response functions as two-column text files, RF_bare_final.txt and RF_sleeve20_final.txt. A README.txt lists the material and density codes used. Simulation output files are available from the corresponding author on request.

## Supplementary Materials

The following supporting information can be downloaded at: https://www.mdpi.com/article/10.3390/s26196266/s1.

## References

[1] Köhli M., Schrön M., Zreda M., Schmidt U., Dietrich P., Zacharias S. (2015). Footprint characteristics revised for field-scale soil moisture monitoring with cosmic-ray neutrons. Water Resour. Res..

[2] Bogena H.R., Schrön M., Jakobi J., Ney P., Zacharias S. (2022). COSMOS-Europe: A European network of cosmic-ray neutron soil moisture sensors. Earth Syst. Sci. Data.

[3] Said H., Mbaye M., Heng L., Fulajtár E., Weltin G., Franz T., Dercon G., Strauss P., Rab G., Al-Menaia H.S. (2021). High-resolution soil moisture mapping through the use of Cosmic-Ray Neutron Sensor and Sentinel-1 data for temperate and semi-arid environments. Front. Water.

[4] Brogi C., Bogena H., Köhli M., Huisman J.A., Hendricks-Franssen H.J., Dombrowski O. (2022). Feasibility of irrigation monitoring with cosmic-ray neutron sensors. Geosci. Instrum. Methods Data Syst..

[5] Brogi C., Pisinaras V., Köhli M., Dombrowski O., Hendricks-Franssen H.J., Babakos K., Chatzi A., Panagopoulos A., Bogena H. (2023). Monitoring Irrigation in Small Orchards with Cosmic-Ray Neutron Sensors. Sensors.

[6] Brogi C., Nieberding F., Scheiffele L., Daccache A., Schrön M., Bogena H. (2026). Irrigation Monitoring with Cosmic-Ray Neutron Sensors: Unraveling Field Experiments with Neutron Transport Simulations. Water Resour. Res..

[7] Li D., Schrön M., Köhli M., Bogena H., Weimar J., Jiménez Bello M.A., Han X., Martínez Gimeno M.A., Zacharias S., Vereecken H. (2019). Can Drip Irrigation be Scheduled with Cosmic-Ray Neutron Sensing?. Vadose Zone J..

[8] Chen X., Song W., Shi Y., Liu W., Lu Y., Pang Z., Chen X. (2022). Application of Cosmic-Ray Neutron Sensor Method to Calculate Field Water Use Efficiency. Water.

[9] Jakobi J., Huisman J.A., Vereecken H., Diekkrüger B., Bogena H. (2018). Cosmic Ray Neutron Sensing for Simultaneous Soil Water Content and Biomass Quantification in Drought Conditions. Water Resour. Res..

[10] Baatz R., Davies P., Nasta P., Bogena H. (2025). Data-driven scaling methods for soil moisture cosmic ray neutron sensors. Hydrol. Earth Syst. Sci..

[11] Heistermann M., Altdorff D., Francke T., Schrön M., Grosse P.M., Markert A., Bauriegel A., Bíró P., Attinger S., Beyrich F. (2026). Soil moisture monitoring with cosmogenic neutrons: An asset for the development and assessment of soil moisture products in the state of Brandenburg (Germany). Nat. Hazards Earth Syst. Sci..

[12] Gazzola E., Marr P., Donato A., Díaz-Delgado R., Colombo N., Gallarate M., Gianessi S., Kanta R., Glade T., Valero A. (2025). Cosmic-Ray Neutron Sensing as a multi-purpose technology in eLTER platforms. ARPHA Conf. Abstr..

[13] Stowell P., Kamienski C., Heideker A., Silva D., Kleinschmidt J.H., Prati R., Chadwick P., Power D., Rosolem R., Köhli M. (2026). COSMIC-SWAMP: IoT Processing of Cosmic-Ray Soil Moisture Sensors. IEEE Access.

[14] Hamdouni A. (2026). Artificial Intelligence-Driven Integrated Water Management and Agricultural Sustainability: Evidence from Saudi Arabia. Resources.

[15] Jabbari A.M.A., Teli T.A., Masoodi F., Reegu F., Uddin M., Albakri A. (2024). Prioritizing factors for the adoption of IoT-based smart irrigation in Saudi Arabia: A GRA/AHP approach. Front. Agron..

[16] Kapkwang C.C., Onyando J., Kundu P., Hoedjes J.C.B. (2021). Evaluation of Spatio-Temporal Soil Moisture Variability in Semi-Arid Rangeland Ecosystem, Maasai Mara National Reserve, Kenya. J. Eng. Res. Rep..

[17] Köhli M., Weimar J. (2026). Beyond Helium-3: Instruments for Cosmic-Ray Neutron Sensing Based on Boron-10 Neutron Detectors. Instruments.

[18] Kouzes R.T., Ely J.H., Erikson L.E., Kernan W.J., Lintereur A.T., Siciliano E.R., Stephens D.L., Stromswold D.C., Van Ginhoven R.M., Woodring M.L. (2010). Neutron detection alternatives to ^3^He for national security applications. Nucl. Instrum. Methods Phys. Res. Sect. A.

[19] Zeitelhack K. (2012). Search for alternative techniques to helium-3 based detectors for neutron scattering applications. Neutron News.

[20] Weimar J., Schattan P., Schrön M., Köhli M., Gugerli R., Stevanato L., Achleitner S., Schmidt U. (2021). A reevaluation of the atmospheric pressure dependence of secondary cosmic-ray neutrons in the context of Cosmic-Ray Neutron Sensing. Front. Water.

[21] Desai S.S., Rao M.N. (2021). Effect of temperature on performance of Boron Trifluoride (BF_3_) gas-based neutron proportional counters. Radiat. Meas..

[22] Wang C., Yu R., Xia W., Gong J. (2025). Advances in High-Temperature Irradiation-Resistant Neutron Detectors. Sensors.

[23] Köhli M., Schrön M., Zacharias S., Schmidt U. (2023). URANOS v1.0—The Ultra Rapid Adaptable Neutron-Only Simulation for Environmental Research. Geosci. Model Dev..

[24] Köhli M., Schrön M., Zacharias S., Schmidt U. (2025). URANOS—A novel voxel engine Neutron Transport Monte-Carlo Simulation. J. Phys. Conf. Ser..

[25] Krane K.S. (1988). Introductory Nuclear Physics.

[26] Simpson J.A. (1951). Change of Cosmic-Ray Neutron Intensity with Altitude and Latitude. Phys. Rev..

[27] Köhli M., Weimar J., Schrön M., Baatz R., Schmidt U. (2021). Soil Moisture and Air Humidity Dependence of the Above-Ground Cosmic-Ray Neutron Intensity. Front. Water.

[28] National Institute for Occupational Safety and Health (2019). NIOSH Pocket Guide to Chemical Hazards: Boron trifluoride.

[29] National Institute for Occupational Safety and Health (1976). Criteria for a Recommended Standard: Occupational Exposure to Boron Trifluoride.

[30] Mashao D., Alton T., Binnersley C., Bradnam S., Croft S., Joyce M.J., Packer L., Turner T., Wild J., Aspinall M. (2025). Boron-coated straw detector efficiency comparison with ^3^He proportional counters—A prospect for cosmic ray neutron monitoring. Nucl. Instrum. Methods Phys. Res. Sect. A.

[31] Mirion Technologies (2024). PN 25/PN 50 B-10 Proportional Counters—Neutron Flux Detectors for the Source Range. Mirion Technologies Product Datasheet. https://assets-mirion.mirion.com/prod-20220822/cms4_mirion/files/pdf/spec-sheets/pn-25-50-data-sheet.pdf.

[32] Baker Hughes/Reuter-Stokes (2024). Boron-10 Lined Proportional Neutron Detectors—Radiation Measurement and Monitoring. Technical Report, Baker Hughes Company Product Datasheet. https://www.bakerhughes.com/sites/bakerhughes/files/2024-01/boron-10_lined_proportional_neutron_detectors.pdf.

[33] Kreusch Filho A. (2025). Progress in the Design of New Gas-Based Neutron Detectors: A Critical Review. Braz. J. Radiat. Sci..

[34] Hafeji R., Raspino D., Schooneveld E., Rhodes N. (2022). Gain measurements of *μ*RWELLs in 3He-CF4 mixtures for thermal neutron detectors. Nucl. Instrum. Methods Phys. Res. Sect. A.

[35] Kim G.H., Lee M., Jung Y., Yoon E., Ahn S. (2024). Simulation of Radiation Damage and He Accumulation Induced by ^10^B(n,*α*)^7^Li Reactions in Al-B_4_C Neutron Absorbers Used in Spent Fuel Pools. Int. J. Energy Res..

[36] Prchlik J., O’Connor D., Merenda K. Proportional Counter Space Charge Simulations. Proceedings of the IEEE Nuclear Science Symposium and Medical Imaging Conference (NSS/MIC).

[37] Tsorbatzoglou K., McKeag R.D. (2011). Novel and efficient ^10^B lined tubelet detector as a replacement for ^3^He neutron proportional counters. Nucl. Instrum. Methods Phys. Res. Sect. A.

[38] StyX Neutronica GmbH (2023). StX S2+ Cosmic Ray Neutron Detector: Technical Reference Manual.

[39] Akhter F., Soomro S.A., Inglezakis V.J. (2021). Silica aerogels: A review of synthesis, applications and fabrication of hybrid composites. J. Porous Mater..

[40] Li H., Xu W., Zhu L., Xiao F., Yu Z., Hao B., Huang W., Zhao K. (2024). Study on thermal insulation performance of silica aerogel thermal insulation blankets. Gels.

[41] Baldinelli G., Asdrubali F., Chiatti C., Gandola D.M., Fantucci S., Serra V., Villamil Cárdenas V., Autretto G., Cottone R., Turrioni C. (2026). Thermal Characterization of Innovative Insulating Materials Through Different Methods: An Intra-Laboratory Study. Sustainability.

[42] Zhu Z., Zhang W., Huang H., Li W., Ling H., Zhang H. (2025). A review of high-temperature resistant silica aerogels: Structural evolution and thermal stability optimization. Gels.

[43] Anderson A.M., Carroll M.K. (2023). Hydrophobic silica aerogels. Springer Handbook of Aerogels.

[44] Aspen Aerogels (2025). Pyrogel XTE/XTF Installation Manual: Recommended Guidelines for Installing Flexible Blanket Insulation.

[45] Fiorini C.V., Merli F., Belloni E., Carroll M.K., Anderson A.M., Buratti C. (2023). Long-term performance of monolithic silica aerogel with different hydrophobicities: Physical and color rendering properties after an accelerated aging process. Gels.

[46] Sato T., Niita K. (2006). Analytical functions to predict cosmic-ray neutron spectra in the atmosphere. Radiat. Res..

[47] Maghrabi A., Alruhaili A., Alzahrani S., Alharbi H., Almasoudi A. (2023). Cosmic ray measurements at high cutoff rigidity site—Preliminary results. J. Atmos. Sol.-Terr. Phys..

[48] Schrön M., Köhli M., Zacharias S. (2023). Signal contribution of distant areas to cosmic-ray neutron sensors—Implications for footprint and sensitivity. Hydrol. Earth Syst. Sci..

[49] Schrön M., Köhli M., Scheiffele L., Iwema J., Bogena H.R., Lv L., Martini E., Baroni G., Rosolem R., Weimar J. (2017). Improving calibration and validation of cosmic-ray neutron sensors in the light of spatial sensitivity. Hydrol. Earth Syst. Sci..

[50] Franz T., Wahbi A., Zhang J., Vreugdenhil M., Heng L., Dercon G., Strauss P., Brocca L., Wagner W. (2020). Practical Data Products From Cosmic-Ray Neutron Sensing for Hydrological Applications. Front. Water.

[51] Köhli M., Schrön M., Schmidt U. (2018). Response functions for detectors in cosmic ray neutron sensing. Nucl. Instrum. Methods Phys. Res. Sect. A.

